# Dynamics of an SEIDW epidemic model for primary amebic meningoencephalitis threshold analysis, bifurcation, and optimal environmental control

**DOI:** 10.3389/fpubh.2026.1851065

**Published:** 2026-06-11

**Authors:** Ramraj G., Poornima T.

**Affiliations:** Department of Mathematics, Vellore Institute of Technology, Vellore, India

**Keywords:** environmental transmission, *Naegleria fowleri*, SEIDW epidemic model, primary amebic meningoencephalitis, optimal control strategies

## Abstract

Primary amebic meningoencephalitis (PAM), caused by the thermophilic amoeba *Naegleria fowleri*, is a rare but almost invariably fatal neurological infection. Unlike classical communicable diseases, PAM transmission is exclusively environmentally driven, with no human-to-human transmission. This paper develops and rigorously analyzes a novel susceptible-exposed-infected-dead-water (SEIDW) epidemic model that explicitly couples human infection dynamics with an environmental reservoir of *N. fowleri*. We establish the fundamental properties of the model including positivity, boundedness, and invariance of solutions. Unlike classical SIR-type models where the basic reproduction number $R_{e}$ derives from human-to-human transmission cycles, the one-directional transmission structure of PAM (environment → human only, with no human-to-environment feedback) renders the standard next-generation matrix approach inapplicable. We instead derive the environmental reproduction number $R_{e}=r_{W}/\left(\delta_{W}+\kappa C_{w}\right)$ via Jacobian eigenvalue analysis and demonstrate that it serves as a sharp threshold parameter: when $R_{e}<1$, the disease-free equilibrium is globally asymptotically stable; when $R_{e}>1$, a unique endemic equilibrium exists and is globally asymptotically stable under biologically plausible conditions. We conduct a complete bifurcation analysis revealing that the model undergoes a forward transcritical bifurcation at $R_{e}=1$. Sensitivity analysis reveals that $R_{e}$ depends exclusively on environmental parameters (*r*_*W*_, δ_*W*_, κ*C*_*w*_), while human parameters (β, σ, α) affect disease burden but not persistence—a finding with profound public health implications. We extend the model to include time-dependent optimal control strategies representing behavioral interventions, environmental sanitation, and personal protective measures. Pontryagin's maximum principle yields characterization of optimal controls, and numerical simulations demonstrate that integrated environmental management is substantially more effective than clinical interventions alone. PRCC and variance-based sensitivity analysis via Latin Hypercube Sampling confirms model robustness under parameter variability. This mathematical framework provides rigorous foundations for designing evidence-based PAM prevention strategies.

## 1 Introduction

### 1.1 Biological background and public health significance

Primary amebic meningoencephalitis (PAM) is a devastating neurological disease caused by the free-living amoeba *Naegleria fowleri*. First identified in 1965 by Australian researchers Malcolm Fowler and Rodney Carter (1), this pathogen has since been recognized as a globally distributed threat. The infection occurs when warm freshwater containing the amoeba enters the nasal cavity, typically during recreational activities such as swimming, diving, or water sports. The trophozoites migrate along the olfactory nerve to the brain, where they cause acute inflammation, hemorrhage, and necrosis of brain tissues (2, 3).

The clinical course of PAM is characterized by rapid progression and devastating outcomes. Initial symptoms—severe headache, fever, nausea, and vomiting often mimic bacterial meningitis, leading to frequent misdiagnosis. Within 3–7 days, patients typically progress to altered mental status, seizures, and coma. Despite the availability of antimicrobial therapies including amphotericin B, miltefosine, and azithromycin, the case fatality rate exceeds 95% (4, 5). From 1962 to 2023, only a handful of survivors have been documented worldwide, making PAM one of the most lethal infectious diseases known (6).

Unlike conventional epidemic diseases such as influenza, COVID-19, or measles, PAM exhibits no human-to-human transmission. The sole reservoir of infection is the environment specifically warm freshwater lakes, rivers, hot springs, and inadequately chlorinated swimming pools (7–9). This unique transmission dynamic necessitates epidemic modeling approaches in which the environment plays a central role. The presence of *N. fowleri* is highly temperature-dependent, with optimal growth between 25 and 45°C and higher prevalence in waters above 25–30°C (10, 11).

**Figure 1:**
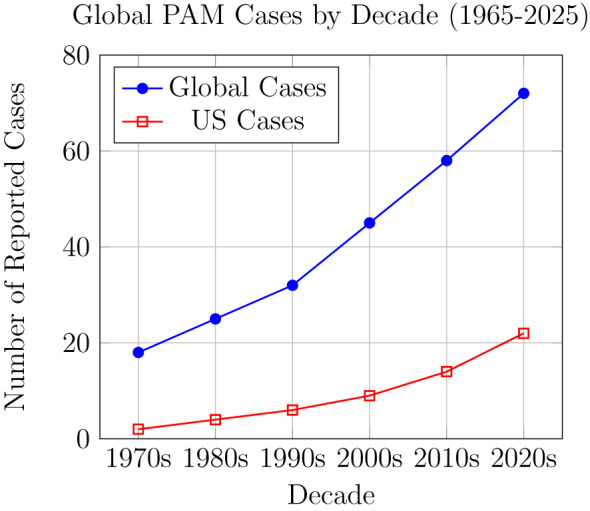
Temporal trend of reported PAM cases globally and in the United States (1965–2025). The increasing trend reflects improved surveillance, climate change, and expanding geographic range of *N. fowleri*.

Figure 1 summarizes the decade-wise increase in reported PAM cases used to contextualize this public health motivation. Climate change, urbanization, and increased recreational water use are expanding the ecological niches suitable for *N. fowleri*. Rising global temperatures extend the duration of warm seasons and the geographic range of suitable habitats (12). Recent cases have been reported in northern US states previously considered non-endemic, and emerging cases in Europe and Asia suggest ongoing geographic expansion (13, 14). These trends raise urgent concerns about increasing PAM risk in previously unaffected regions.

### 1.2 Epidemiological characteristics and transmission dynamics

The regional distribution of reported PAM cases is summarized in Table 1. The epidemiology of PAM is characterized by several distinctive features that distinguish it from typical infectious diseases:

(i) Environmental reservoir dominance: the pathogen exists primarily in environmental water bodies, with human cases representing incidental infections rather than necessary steps in the pathogen's life cycle.
(ii) Seasonal pattern: cases peak during summer months when water temperatures are highest, with approximately 80% of US cases occurring between July and September (5).
(iii) Age distribution: PAM predominantly affects children and young adults, reflecting age-related recreational water exposure patterns rather than immunological factors.
(iv) Geographic clustering: cases concentrate in regions with warm freshwater bodies, including the southern United States (Texas, Florida, Arizona), Australia, Pakistan, and India (15).
(v) Rarity with high fatality: despite low incidence, the near-certain mortality makes PAM a critical public health concern wherever warm freshwater recreation occurs.

**Table 1:** Reported distribution of primary amebic meningoencephalitis (PAM) cases by region (1962–2025).

Region	Reported cases	Percentage	Key characteristics
United States	165	43	Highest concentration in southern states
India	80	21	Kerala accounts for 90% of Indian cases
Pakistan	25	7	Increasing trend in recent decades
Australia	22	6	Northern regions endemic
Mexico	15	4	Central and southern regions
Other regions	74	19	Sporadic cases worldwide
**Global total**	**381**	**100**	**Underreporting likely significant**

Bold values indicate the globally dominant category (United States, 43% of reported cases) used as the primary reference point for regional comparison.

### 1.3 Mathematical epidemiology of environmentally transmitted diseases

From a mathematical epidemiology perspective, most infectious disease models have focused on directly transmitted pathogens (17–20). The classical Susceptible-Infected-Recovered (SIR) framework and its extensions have provided fundamental insights into disease dynamics, including threshold conditions for outbreaks, herd immunity, and the effects of vaccination. However, these models assume direct human-to-human transmission and do not adequately capture diseases where environmental reservoirs dominate.

Environmentally transmitted diseases require fundamentally different modeling approaches (21–23). Key distinctions include:

1. Force of infection depends on environmental pathogen concentration rather than infected human population.
2. Reservoir dynamics follow their own ecological laws (growth, decay, and carrying capacity).
3. Control strategies must target environmental reservoirs rather than human-to-human transmission chains.
4. Threshold conditions incorporate both human epidemiological factors and environmental persistence.

Models incorporating environmental reservoirs have been developed for various diseases including cholera (25), legionellosis (21), and schistosomiasis (24). These models reveal distinct threshold conditions and control implications, often emphasizing ecological interventions over clinical ones. The inclusion of environmental components introduces additional non-linearities and potentially richer dynamical behavior, including bistability, hysteresis, and periodic oscillations (26).

Of particular relevance to the present work are models incorporating environmental water reservoirs for waterborne pathogens. Codeço (39) introduced the SIR-B model for cholera in which bacteria in the aquatic reservoir drive transmission, but humans shed bacteria back into the environment, creating a cyclic transmission loop. Tien and Earn (40) extended this to an SIWR framework allowing both direct (person-to-person) and indirect (water-to-person) transmission pathways. More recently, Ghosh et al.(31) combined mechanistic cholera modeling with machine learning for outbreak forecasting, and Wang and Feng (34) analyzed global dynamics of waterborne pathogen models under environmental pollution. Optimal control approaches for environmentally transmitted diseases have been studied alongside epidemic models incorporating comorbidity and non-pharmaceutical interventions (29, 30, 32). Seasonal and spatial extensions of waterborne models have also been studied (35). Recent advances in *N. fowleri* epidemiology include comprehensive global reviews (36, 38) and calls for One Health surveillance frameworks (37).

A critical distinction separates these models from the PAM context. In cholera-type models, infected humans contribute to the environmental reservoir through fecal shedding, creating a closed human↔environment transmission cycle. For PAM, however, *N. fowleri* does not require human hosts for its life cycle; human infection is a dead-end for the pathogen (Assumption 0.0.5). This one-directional transmission structure (environment → human only) leads to fundamentally different threshold dynamics, as we demonstrate in Section 6. Table 2 summarizes these structural differences.

**Table 2:** Structural comparison of SEIDW with existing environmental epidemic models.

Feature	SIR-B (39)	SIWR (40)	Cholera (31)	SEIDW (Ours)
Target disease	Cholera	General WBD	Cholera	PAM
Human → human	No	Yes	Yes	No
Human → water feedback	Yes	Yes	Yes	**No**
Exposed compartment	No	No	No	**Yes**
Death compartment	No	No	No	**Yes**
Recovery	Yes	Yes	Yes	**No (>95% CFR)**
Transmission cycle	Cyclic	Cyclic	Cyclic	**One-way**
$R_{e}$ incl. human params	Yes	Yes	Yes	**No**
Optimal control	Some	No	ML-based	**Yes (Pontryagin)**
Bifurcation analysis	Limited	No	No	**Yes**

Bold values in the SEIDW column indicate distinguishing structural features unique to the present model: absence of human-to-water feedback, inclusion of exposed (E) and death (D) compartments, one-way transmission cycle, and exclusion of human parameters from the reproduction number — all of which differentiate the SEIDW model from prior waterborne disease models.

### 1.4 Mathematical contributions and novelty

This paper develops a rigorous mathematical framework for PAM transmission dynamics that addresses the unique characteristics of environmentally transmitted diseases. Our contributions include:

(i) Novel SEIDW compartmental structure: we introduce a susceptible-exposed-infected-dead-water (SEIDW) model that explicitly couples human population dynamics with an environmental reservoir of *N. fowleri*, incorporating both exposed (asymptomatic) and dead compartments that are clinically relevant for PAM.
(ii) Complete threshold analysis: we derive the environmental reproduction number $R_{e}$ via Jacobian eigenvalue analysis, demonstrating that the standard next-generation matrix method yields a degenerate result for one-directional transmission models, and establish $R_{e}$ as a sharp threshold parameter for disease persistence and extinction.
(iii) Global stability results: using Lyapunov function techniques, we prove global asymptotic stability of the disease-free equilibrium when $R_{e}<1$ and establish conditions for global stability of the endemic equilibrium when $R_{e}>1$.
(iv) Bifurcation analysis: we demonstrate that the model undergoes a forward transcritical bifurcation at $R_{e}=1$, ruling out subcritical (backward) bifurcations under biologically reasonable parameter assumptions.
(v) Comprehensive sensitivity analysis: we compute normalized forward sensitivity indices and PRCC via Latin Hypercube Sampling, revealing that $R_{e}$ depends exclusively on environmental parameters while disease burden depends on both environmental and human parameters.
(vi) Optimal control formulation: we extend the model to include three time-dependent control interventions and derive optimal control strategies using Pontryagin's maximum principle.
(vii) Rigorous numerical validation: we conduct extensive numerical simulations and Monte Carlo uncertainty analysis to validate theoretical results and assess model robustness.

### 1.5 Paper organization

The remainder of this paper is organized as follows. Section 2 presents the SEIDW model formulation, including biological justification of assumptions and parameter definitions. Section 3 establishes fundamental properties of the model including positivity, boundedness, and invariant regions. Section 4 introduces non-dimensionalization to reduce parameter complexity and simplify analysis. Section 5 provides complete equilibrium analysis, including existence conditions for disease-free and endemic equilibria. Section 6 derives the basic reproduction number and establishes its threshold properties. Section 7 presents global stability analysis using Lyapunov functions. Section 8 investigates bifurcation behavior, including transcritical bifurcation and sensitivity analysis. Section 9 extends the model to include optimal control strategies. Section 10 presents numerical simulations and uncertainty analysis. Section 11 concludes with discussion of public health implications and future research directions.

## 2 Model formulation

### 2.1 Biological assumptions and compartmental structure

We consider the human population divided into four distinct epidemiological compartments based on infection status and clinical progression. This compartmental structure is designed to capture the unique features of PAM pathogenesis:

1. Susceptible (*S*(*t*)): individuals who have not been infected and are at risk of infection through exposure to contaminated water. The susceptible population is replenished through recruitment (births and immigration) at rate Λ and depleted through natural mortality at rate μ and infection.
2. Exposed (*E*(*t*)): individuals who have been infected but are not yet symptomatic. In PAM, the incubation period typically ranges from 1 to 9 days (mean 5 days) before symptom onset. During this period, individuals are not infectious (as there is no human-to-human transmission) but represent the pool from which symptomatic cases emerge.
3. Infected (*I*(*t*)): symptomatic individuals with active PAM. These individuals progress rapidly to death, with mean time from symptom onset to death of approximately 5 days. There is no recovery in the classical sense; survivors are exceptionally rare.
4. Deceased (*D*(*t*)): cumulative deaths from PAM. While this compartment does not affect transmission dynamics, it is included for public health monitoring and to capture disease burden.

The environmental component is represented by:

(W) Water reservoir (*W*(*t*)): the concentration (or density) of viable *N. fowleri* amoebae in recreational water bodies. This reservoir is the sole source of infection. The dynamics of *W* are governed by ecological processes including growth, competition (modeled through carrying capacity), natural mortality, and human-mediated clearance.

### 2.2 Key model assumptions

The following biological assumptions underpin our model formulation:

** Assumption 0.0.1 (No human-to-human transmission)**. PAM cannot be transmitted directly between humans. All infections result from exposure to contaminated water. This is supported by extensive epidemiological evidence showing no documented cases of person-to-person transmission.

** Assumption 0.0.2 (No recovery)**. Given the >95% case fatality rate and the absence of effective treatments that reliably produce recovery, we assume that all symptomatic infected individuals eventually die from the disease. The negligible number of survivors is not sufficient to warrant a recovered compartment.

** Assumption 0.0.3 (Environmental pathogen dynamics)**. The environmental amoeba population follows logistic growth in the absence of control, reflecting resource limitations and intraspecific competition. This is a standard ecological modeling assumption that captures the carrying capacity of the environment.

** Assumption 0.0.4 (Constant recruitment)**. The susceptible population is replenished at a constant rate Λ, representing births and immigration. This simplification is appropriate for the relatively short time scales considered in outbreak analysis.

** Assumption 0.0.5 (No feedback from humans to environment)**. Human cases do not contribute significantly to the environmental reservoir of *N. fowleri*. The amoeba does not require human hosts for reproduction or persistence, and human infection is a dead-end for the pathogen.

### 2.3 Governing differential equations

Based on the above assumptions, the SEIDW model is described by the following system of ordinary differential equations:


$$\begin{array}{l} \frac{dS}{dt}=\text{Λ}-\beta cWS-\mu S, \end{array}$$ (1)

$$\begin{array}{l} \frac{dE}{dt}=\beta cWS-\left(\sigma +\mu \right)E, \end{array}$$ (2)

$$\begin{array}{l} \frac{dI}{dt}=\sigma E-\left(\gamma +\mu +\alpha \right)I, \end{array}$$ (3)

$$\begin{array}{l} \frac{dD}{dt}=\alpha I, \end{array}$$ (4)

$$\begin{array}{l} \frac{dW}{dt}=r_{W}W\left(1-\frac{W}{K}\right)-\delta_{W}W-\kappa C_{w}\left(t\right)W. \end{array}$$ (5)

### 2.4 Parameter definitions and biological interpretation

All parameters are nonnegative and have clear biological interpretations, as summarized in Table 3:

**Table 3:** Key parameters of the SEIDW model with biological interpretation.

Symbol	Description	Typical range
Λ	Recruitment rate of susceptible individuals	0.1–10 day^−1^
μ	Natural mortality rate (excluding PAM)	3.4 × 10^−5^ day^−1^
β	Transmission coefficient per amoeba concentration	0.01–1 day^−1^ (L^−1^)
*c*	Contact rate with contaminated water	0.001–0.1 dimensionless
σ	Rate of progression from *E* to *I*	0.1–0.5 day^−1^
α	Disease-induced death rate	0.1–0.5 day^−1^
γ	Clinical removal rate (hospitalization)	0.05–0.2 day^−1^
*r*_*W*_	Pathogen growth rate in water	0.05–0.5 day^−1^
*K*	Environmental carrying capacity	10^2^–10^6^ L^−1^
δ_*W*_	Natural decay rate of pathogen	0.01–0.1 day^−1^
κ	Environmental control effectiveness	0.1–1 dimensionless
*C*_*w*_	Environmental control intensity	0–1 dimensionless

The force of infection λ(*t*) = β*cW*(*t*) represents the per-capita rate at which susceptible individuals become infected. This formulation assumes that infection risk is proportional to environmental pathogen concentration and contact frequency.

### 2.5 Schematic representation

**Figure 2:**
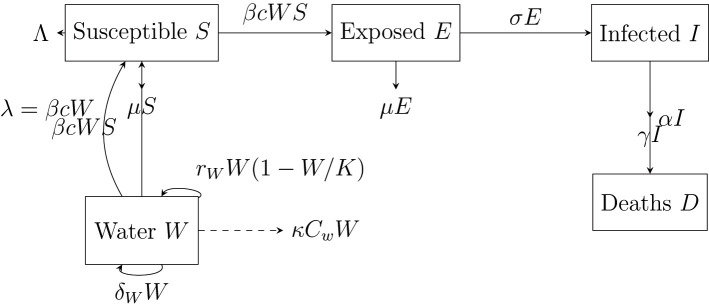
Schematic diagram of the SEIDW model for PAM. Solid arrows represent transitions between compartments; dashed arrows indicate control interventions.

The compartmental flow corresponding to Equations 1–5 is shown in Figure 2.

## 3 Basic properties of the model

### 3.1 Positivity of solutions

** Theorem 3.1** (Positivity). For any initial conditions *S*(0) ≥ 0, *E*(0) ≥ 0, *I*(0) ≥ 0, *D*(0) ≥ 0, *W*(0) ≥ 0, the solutions of system Equation 1–5 remain non-negative for all *t* ≥ 0.

Proof.We prove positivity component-wise. From the Equations 6–10 consider the susceptible compartment.


$$\begin{array}{l} \frac{dS}{dt}=\text{Λ}-\left(\beta cW+\mu \right)S\geq -\left(\beta cW+\mu \right)S. \end{array}$$ (6)

This inequality yields $S\left(t\right)\geq S\left(0\right)\exp\left(-\int_{0}^{t}\left(\beta cW\left(\tau \right)+\mu \right)d\tau \right)\geq 0$.

For the exposed compartment, using Equation 2,


$$\begin{array}{l} \frac{dE}{dt}=\beta cWS-\left(\sigma +\mu \right)E\geq -\left(\sigma +\mu \right)E, \end{array}$$ (7)

so *E*(*t*) ≥ *E*(0)*e*^−(σ+μ)*t*^ ≥ 0.

Similarly, for the infected compartment,


$$\begin{array}{l} \frac{dI}{dt}=\sigma E-\left(\gamma +\mu +\alpha \right)I\geq -\left(\gamma +\mu +\alpha \right)I, \end{array}$$ (8)

giving *I*(*t*) ≥ *I*(0)*e*^−(γ+μ+α)*t*^ ≥ 0.

The death compartment accumulates non-negative contributions:


$$\begin{array}{l} \frac{dD}{dt}=\alpha I\geq 0, \end{array}$$ (9)

so *D*(*t*) is monotone non-decreasing and non-negative if *D*(0) ≥ 0.

For the environmental reservoir, from Equation 5,


$$\begin{array}{l} \frac{dW}{dt}=W\left(r_{W}\left(1-\frac{W}{K}\right)-\delta_{W}-\kappa C_{w}\right). \end{array}$$ (10)

If *W*(0) = 0, then Ẇ = 0, so *W*(*t*) = 0 for all *t*. If *W*(0) > 0, suppose there exists a first time *t*_1_ > 0 such that *W*(*t*_1_) = 0 and *W*(*t*) > 0 for *t*∈[0, *t*_1_). Then Ẇ(*t*_1_) = 0, contradicting the assumption that *t*_1_ is the first zero. Hence *W*(*t*) > 0 for all *t* when *W*(0) > 0.

### 3.2 Boundedness of solutions

** Theorem 3.2** (Boundedness of Human Population). The total human population *N*(*t*) = *S*(*t*)+*E*(*t*)+*I*(*t*)+*D*(*t*) satisfies the Equations 11–16


$$\begin{array}{l} \underset{t\to \infty }{lim sup}N\left(t\right)\leq \frac{\text{Λ}}{\mu }, \end{array}$$ (11)

and is uniformly bounded.

Proof. Summing Equations 1–4 yields


$$\begin{array}{l} \frac{dN}{dt}=\text{Λ}-\mu N-\alpha I-\gamma I\leq \text{Λ}-\mu N. \end{array}$$ (12)

Applying the standard comparison theorem, we obtain


$$\begin{array}{l} N\left(t\right)\leq N\left(0\right)e^{-\mu t}+\frac{\text{Λ}}{\mu }\left(1-e^{-\mu t}\right). \end{array}$$ (13)

Taking the limit superior as *t* → ∞ gives the desired result. The solution *N*(*t*) is uniformly bounded since it is bounded above by max{*N*(0), Λ/μ}.

** Theorem 3.3** (Boundedness of Environmental Reservoir). The environmental variable *W*(*t*) satisfies


$$\begin{array}{l} \underset{t\to \infty }{lim sup}W\left(t\right)\leq K{\left(1-\frac{\delta_{W}+\kappa C_{w}}{r_{W}}\right)}_{+}, \end{array}$$ (14)

where (*x*)_+_ = max{0, *x*}.

Proof. From Equation 5, we have


$$\begin{array}{l} \frac{dW}{dt}\leq r_{W}W\left(1-\frac{W}{K}\right)-\left(\delta_{W}+\kappa C_{w}\right)W. \end{array}$$ (15)

The right-hand side is a logistic equation with modified carrying capacity. Standard results from population dynamics yield that *W*(*t*) is bounded above by the positive equilibrium


$$\begin{array}{l} W_{\max}=K\left(1-\frac{\delta_{W}+\kappa C_{w}}{r_{W}}\right) \end{array}$$ (16)

when *r*_*W*_ > δ_*W*_+κ*C*_*w*_, and decays to zero otherwise.

### 3.3 Invariant region

Define the biologically feasible region (Equation 17):


$$\begin{array}{l} \text{Ω}=\{(S,E,I,D,W)\in {\mathbb{R}}_{+}^{5}:S+E+I+D\leq \frac{\text{Λ}}{\mu },\;\\ W\leq K{\left(1-\frac{\delta_{W}+\kappa C_{w}}{r_{W}}\right)}_{+}\}. \end{array}$$ (17)

** Theorem 3.4** (Forward Invariance). The region Ω is positively invariant for system Equations 1–5.

Proof.The boundary of Ω consists of non-negative coordinate hyperplanes and the upper bounds. We have already shown that solutions starting in ${\mathbb{R}}_{+}^{5}$ remain non-negative. For the upper bound on *N*, if *N*(*t*) = Λ/μ, then Ṅ ≤ 0. For the upper bound on *W*, similar reasoning applies. Thus, trajectories cannot leave Ω.

## 4 Non-dimensionalization

To reduce the number of parameters and simplify analysis, we introduce dimensionless variables and parameters.

### 4.1 Dimensionless variables

Define:


$$\begin{array}{l} s=\frac{S}{N^{*}},\;e=\frac{E}{N^{*}},\;i=\frac{I}{N^{*}},\;d=\frac{D}{N^{*}},\;w=\frac{W}{K},\;\tau =\mu t, \end{array}$$ (18)

where N^*^ = Λ/μ is the equilibrium total population in the absence of disease. The dimensionless variables are defined in Equations 17, 18.

### 4.2 Dimensionless parameters

Let:


$$\begin{array}{l} \beta_{0}=\frac{\beta cK}{\mu },\;\sigma_{0}=\frac{\sigma }{\mu },\;\gamma_{0}=\frac{\gamma }{\mu },\;\alpha_{0}=\frac{\alpha }{\mu }, \end{array}$$ (19)

$$\begin{array}{l} r=\frac{r_{W}}{\mu },\;\delta =\frac{\delta_{W}}{\mu },\;\kappa_{0}=\frac{\kappa C_{w}}{\mu }. \end{array}$$ (20)

The dimensionless parameters are given in Equations 19, 20.

### 4.3 Dimensionless system

Substituting these transformations into Equations 1–5 yields the dimensionless system (Equations 21–24):


$$\begin{array}{l} \frac{ds}{d\tau }=1-s-\beta_{0}ws, \end{array}$$ (21)

$$\begin{array}{l} \frac{de}{d\tau }=\beta_{0}ws-\left(\sigma_{0}+1\right)e, \end{array}$$ (22)

$$\begin{array}{l} \frac{di}{d\tau }=\sigma_{0}e-\left(\gamma_{0}+1+\alpha_{0}\right)i, \end{array}$$ (23)

$$\begin{array}{l} \frac{dw}{d\tau }=rw\left(1-w\right)-\delta w-\kappa_{0}w, \end{array}$$ (24)

where we have simplified the logistic term using the dimensionless carrying capacity of 1.

### 4.4 Reduced system

Since the death compartment *d* does not affect the dynamics of other variables, we consider the reduced system:


$$\begin{array}{l} \frac{ds}{d\tau }=1-s-\beta_{0}ws, \end{array}$$ (25)

$$\begin{array}{l} \frac{de}{d\tau }=\beta_{0}ws-Ae, \end{array}$$ (26)

$$\begin{array}{l} \frac{di}{d\tau }=\sigma_{0}e-Bi, \end{array}$$ (27)

$$\begin{array}{l} \frac{dw}{d\tau }=rw\left(1-w\right)-\delta w-\kappa_{0}w, \end{array}$$ (28)

where *A* = σ_0_+1 and *B* = γ_0_+1+α_0_.

## 5 Equilibrium analysis

### 5.1 Disease-free equilibrium

The disease-free equilibrium (DFE) is obtained by setting *e* = *i* = 0 and *w* = 0. From Equation 25, we obtain *s* = 1. The disease-free equilibrium is defined in Equations 29–39


$$\begin{array}{l} E_{0}=\left(s,e,i,w\right)=\left(1,0,0,0\right). \end{array}$$ (29)

### 5.2 Environmental equilibrium

The environmental Equation 28 decouples from the human compartments. Setting ẇ = 0 yields the environmental equilibrium is obtained from Equations 30–32:


$$\begin{array}{l} w\left[r\left(1-w\right)-\delta -\kappa_{0}\right]=0. \end{array}$$ (30)

Thus, either *w* = 0 or


$$\begin{array}{l} w^{*}=1-\frac{\delta +\kappa_{0}}{r}. \end{array}$$ (31)

A positive environmental equilibrium exists if and only if *r* > δ+κ_0_.

**Lemma 5.1 (Environmental Persistence)**. The environmental reservoir *w* converges to:

- *w* = 0 if *r* ≤ δ+κ_0_,
- $w^{*}=1-\left(\delta +\kappa_{0}\right)/r$ if *r* > δ+κ_0_.

Proof. Equation 28 is a logistic differential equation with net growth rate *r*−δ−κ_0_. Standard analysis of logistic growth yields the stated convergence.

### 5.3 Endemic equilibrium

When *r* > δ+κ_0_, we have w^*^ > 0. Setting the right-hand sides of Equations 25–27 to zero gives:

From Equation 26:


$$\begin{array}{l} e^{*}=\frac{\beta_{0}w^{*}s^{*}}{A}. \end{array}$$ (32)

From Equation 27:


$$\begin{array}{l} i^{*}=\frac{\sigma_{0}e^{*}}{B}=\frac{\beta_{0}\sigma_{0}w^{*}s^{*}}{AB}. \end{array}$$ (33)

From Equation 25:


$$\begin{array}{l} 0=1-s^{*}-\beta_{0}w^{*}s^{*}\;\Rightarrow \;s^{*}=\frac{1}{1+\beta_{0}w^{*}}. \end{array}$$ (34)

Thus, a unique endemic equilibrium E^*^ = (s^*^, e^*^, i^*^, w^*^) exists when w^*^ > 0, i.e., when *r* > δ+κ_0_. The endemic equilibrium and threshold conditions are given in Equations 33–39.

** Theorem 3.5** (Existence of Endemic Equilibrium). System Equations 25–28 admits a unique endemic equilibrium if and only if *r* > δ+κ_0_.

## 6 Environmental reproduction number and threshold dynamics

### 6.1 Threshold parameter for environmentally driven diseases

For diseases with direct human-to-human transmission, the basic reproduction number $R_{e}$ represents the expected number of secondary infections from a single infected individual and is typically derived via the next-generation matrix (NGM) method (27). However, the SEIDW model for PAM possesses a fundamentally different transmission structure: the environmental reservoir *w* evolves independently of the human compartments (Assumption 0.0.5), and there is no feedback from infected humans to the environment. Consequently, the pathway *w*→*e*→*i*→*d* is one-directional, and no generational transmission cycle exists.

In such models, the standard NGM approach yields a degenerate (nilpotent) next-generation matrix with spectral radius zero, because infected humans cannot generate new environmental contamination. Following the framework of Heesterbeek and Roberts (33) for models where the classical $R_{e}$ is inapplicable, we define the *environmental reproduction number*:


$$\begin{array}{l} R_{e}=\frac{r}{\delta +\kappa_{0}} \end{array}$$ (35)

which represents the ratio of the pathogen net growth rate to its total clearance rate in the environment. This quantity determines whether the environmental reservoir can sustain a viable pathogen population.

** Remark 6.1** (Why the standard NGM fails). Applying the NGM method with infected compartments (*e, i, w*) yields


$$K=FV^{-1}=\left(\begin{array}{ccc} 0 & 0 & \beta_{0}/\left(\delta +\kappa_{0}-r\right)\\ 0 & 0 & 0\\ 0 & 0 & 0 \end{array}\right),$$

which satisfies *K*^2^ = 0 (nilpotent), so ρ(*K*) = 0. The non-zero entry *K*_13_ measures new human infections per unit pathogen, but these infections produce zero new pathogen units, terminating the “generation” chain. The threshold is therefore governed not by the spectral radius of *K* but by the stability of the environmental subsystem itself.

### 6.2 Derivation via Jacobian Eigenvalue analysis

The Jacobian matrix of the reduced system Equations 25–28 evaluated at the DFE *E*_0_ = (1, 0, 0, 0) is:


$$\begin{array}{l} J\left(E_{0}\right)=\left(\begin{array}{cccc} -1 & 0 & 0 & -\beta_{0}\\ 0 & -A & 0 & \beta_{0}\\ 0 & \sigma_{0} & -B & 0\\ 0 & 0 & 0 & r-\left(\delta +\kappa_{0}\right) \end{array}\right). \end{array}$$ (36)

This matrix is block-triangular. The upper-left 3 × 3 block has eigenvalues λ_1_ = −1, λ_2_ = −*A* < 0, and λ_3_ = −*B* < 0, which are always negative. The remaining eigenvalue is:


$$\begin{array}{l} {\text{λ}}_{4}=r-\left(\delta +\kappa_{0}\right)=\left(\delta +\kappa_{0}\right)\left(R_{e}-1\right). \end{array}$$ (37)

** Theorem 6.2** (Threshold Behavior). The disease-free equilibrium *E*_0_ is locally asymptotically stable if $R_{e}<1$ and unstable if $R_{e}>1$.

Proof. All eigenvalues of *J*(*E*_0_) have negative real parts if and only if λ_4_ < 0, which holds precisely when *r* < δ+κ_0_, i.e., $R_{e}<1$.

### 6.3 Biological interpretation

The environmental reproduction number factorizes as:


$$\begin{array}{l} R_{e}=\frac{r_{W}}{\delta_{W}+\kappa C_{w}}=\frac{\text{pathogen growth rate}}{\text{natural decay}+\text{control-induced clearance}}. \end{array}$$ (38)

A key consequence is that *human epidemiological parameters* (β, σ, γ, α) do not affect the persistence threshold. This reflects the biology of PAM: since *N. fowleri* does not depend on human hosts for survival, the amoeba persists or perishes based entirely on environmental conditions. Human parameters affect the *severity* of disease (peak infections, cumulative deaths) but not its *persistence*. This distinction, confirmed by our PRCC sensitivity analysis (Section 11), has profound implications for public health: environmental management is not merely preferable but *necessary and sufficient* for PAM elimination.

### 6.4 Role of human parameters in disease burden

Although $R_{e}$ is independent of human parameters, the endemic infection level is not. When $R_{e}>1$, the endemic infected proportion is:


$$\begin{array}{l} i^{*}=\frac{\beta_{0}\sigma_{0}w^{*}}{AB\left(1+\beta_{0}w^{*}\right)},\;w^{*}=1-\frac{1}{R_{e}}. \end{array}$$ (39)

Thus, β_0_ and σ_0_ modulate the attack rate once the pathogen is established, and α determines the case fatality rate. Reducing human exposure (β*c*) lowers disease burden even when it cannot eliminate the pathogen.

## 7 Global stability analysis

### 7.1 Global stability of disease-free equilibrium

** Theorem 7.1** (Global Stability of DFE). If $R_{e}<1$, the disease-free equilibrium *E*_0_ is globally asymptotically stable in Ω.

Proof.Since the environmental Equation 28 decouples from the human compartments, we analyze its stability separately. Consider the Lyapunov function *L*_*w*_ = *w*. Along solutions of Equation 28:


$${\dot{L}}_{w}=rw\left(1-w\right)-\left(\delta +\kappa_{0}\right)w=w\left[\left(\delta +\kappa_{0}\right)\left(R_{e}-1\right)-rw\right].$$

When $R_{e}<1$, the term $\left(\delta +\kappa_{0}\right)\left(R_{e}-1\right)<0$, so ${\dot{L}}_{w}\leq w\left(\delta +\kappa_{0}\right)\left(R_{e}-1\right)<0$ for all *w* > 0. The set $\left\{{\dot{L}}_{w}=0\right\}$ contains only *w* = 0. By LaSalle's invariance principle, *w*(*t*) → 0 as *t* → ∞.

With *w*(*t*) → 0, the human subsystem becomes asymptotically autonomous:


$$ṡ\to 1-s,\;ė\to -Ae,\;\dot{i}\to \sigma_{0}e-Bi.$$

Since *A* > 0 and *B* > 0, we have *e*(*t*) → 0 and *i*(*t*) → 0 exponentially, while *s*(*t*) → 1. Therefore, all trajectories in Ω converge to *E*_0_.

### 7.2 Global stability of endemic equilibrium

** Theorem 7.2** (Global Stability of Endemic Equilibrium). If $R_{e}>1$, the endemic equilibrium E^*^ = (s^*^, e^*^, i^*^, w^*^) is globally asymptotically stable in the interior of Ω.

Proof.Since the environmental equation decouples, we first establish *w*(*t*) → w^*^. For $R_{e}>1$, Equation 28 is a logistic ODE with positive carrying capacity $w^{*}=1-\left(\delta +\kappa_{0}\right)/r>0$. Standard logistic analysis gives *w*(*t*) → w^*^ for any *w*(0) > 0.

With *w* = w^*^, the human subsystem at equilibrium satisfies:


$$\begin{array}{l} 1=s^{*}\left(1+\beta_{0}w^{*}\right),\;Ae^{*}=\beta_{0}w^{*}s^{*},\;Bi^{*}=\sigma_{0}e^{*}. \end{array}$$ (40)

Consider the Volterra-type Lyapunov function:


$$\begin{array}{l} V_{h}=\left(s-s^{*}-s^{*}\ln\;\frac{s}{s^{*}}\right)+\left(e-e^{*}-e^{*}\ln\;\frac{e}{e^{*}}\right)\\ \;+\frac{A}{\sigma_{0}}\left(i-i^{*}-i^{*}\ln\;\frac{i}{i^{*}}\right). \end{array}$$ (41)

Differentiating and substituting the equilibrium conditions Equation 25, using $1=s^{*}\left(1+\beta_{0}w^{*}\right)$ and $Ae^{*}=\beta_{0}w^{*}s^{*}$:


$$\begin{array}{l} {\dot{V}}_{h}=\left(1+\beta_{0}w^{*}\right)s^{*}\left(2-\frac{s}{s^{*}}-\frac{s^{*}}{s}\right)\\ \;+\beta_{0}w^{*}s^{*}\left(\frac{s}{s^{*}}+\frac{s^{*}e}{se^{*}}-1-\frac{e}{e^{*}}\right). \end{array}$$ (42)

By the arithmetic-geometric mean inequality (*x*+1/*x* ≥ 2 for *x* > 0):

- (2−*s*/s^*^−s^*^/*s*) = −(*s*/s^*^−1)2·s^*^/*s* ≤ 0;
- (*s*/s^*^+s^*^e/(se^*^)−1−*e*/e^*^) ≤ 0 by AM-GM.

Thus ${\dot{V}}_{h}\leq 0$, with equality only when *s* = s^*^ and *e* = e^*^, which forces *i* = i^*^. By LaSalle's invariance principle applied to the product system (*V*_*h*_, *w*), the endemic equilibrium is globally asymptotically stable.

## 8 Bifurcation analysis

### 8.1 Transcritical bifurcation

**Figure 3:**
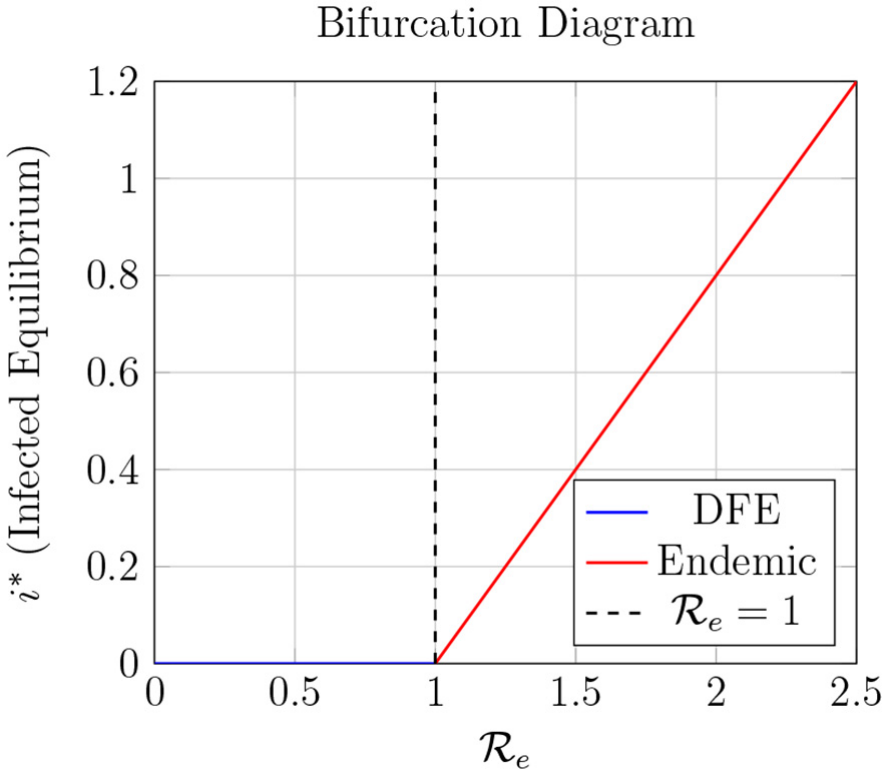
Bifurcation diagram showing forward transcritical bifurcation at $R_{e}=1$. The disease-free equilibrium (blue line) loses stability at $R_{e}=1$, giving rise to a stable endemic equilibrium (red curve).

We investigate the bifurcation behavior as $R_{e}$ passes through unity. This forward transcritical transition is illustrated in Figure 3.

** Theorem 8.1 (Forward Transcritical Bifurcation)**. At $R_{e}=1$, system Equations 25–28 undergoes a forward (supercritical) transcritical bifurcation.

Proof. We use *r* as the bifurcation parameter. At $R_{e}=1$, the critical value is $r^{*}=\delta +\kappa_{0}$. The Jacobian *J*(*E*_0_) at *r* = r^*^ has a simple zero eigenvalue λ_4_ = 0. The right eigenvector **v** satisfying *J*(*E*_0_)**v** = 0 is:


$$\begin{array}{l} \text{v}={\left(-\frac{\beta_{0}}{AB}v_{4},\frac{\beta_{0}}{A}v_{4},\frac{\beta_{0}\sigma_{0}}{AB}v_{4},v_{4}\right)}^{T},\;v_{4}>0. \end{array}$$ (43)

The left eigenvector yields **w** = (0, 0, 0, 1).

Following Castillo-Chavez and Song (28), since *w*_*k*_≠0 only for *k* = 4, and *f*_4_ = *rw*(1−*w*)−(δ+κ_0_)*w*:

For coefficient *a*: $\partial^{2}f_{4}/\partial w^{2}=-2r$, giving $a=v_{4}^{2}\cdot \left(-2r^{*}\right)=-2\left(\delta +\kappa_{0}\right)v_{4}^{2}<0$.

For coefficient *b*: $\partial^{2}f_{4}/\left(\partial w\partial r\right)=1-2w$, evaluated at *w* = 0 gives 1, so *b* = *v*_4_ > 0.

Since *a* < 0 and *b* > 0, the bifurcation is forward (supercritical). The endemic equilibrium exists for $R_{e}>1$ and is locally stable.

### 8.2 Sensitivity analysis

To quantify the relative importance of parameters in determining $R_{e}$, we compute normalized forward sensitivity indices.

** Definition 8.2** (Normalized Forward Sensitivity Index). The normalized forward sensitivity index of $R_{e}$ with respect to a parameter *p* is defined as Equations 41–47:


$$\begin{array}{l} {\text{Γ}}_{p}^{R_{e}}=\frac{\partial R_{e}}{\partial p}\cdot \frac{p}{R_{e}}. \end{array}$$ (44)

** Theorem 8.3** (Sensitivity Indices). The normalized forward sensitivity indices of $R_{e}=r/\left(\delta +\kappa_{0}\right)$ are:


$$\begin{array}{l} {\text{Γ}}_{r}^{R_{e}}=+1, \end{array}$$ (45)

$$\begin{array}{l} {\text{Γ}}_{\delta }^{R_{e}}=-\frac{\delta }{\delta +\kappa_{0}}, \end{array}$$ (46)

$$\begin{array}{l} {\text{Γ}}_{\kappa_{0}}^{R_{e}}=-\frac{\kappa_{0}}{\delta +\kappa_{0}}. \end{array}$$ (47)

All human parameters (β_0_, σ_0_, γ_0_, α_0_) have zero sensitivity with respect to $R_{e}$. The baseline sensitivity values implied by this result are reported in Table 4.

**Table 4:** Sensitivity indices for $R_{e}$ at baseline values (*r*_*W*_ = 0.15, δ_*W*_ = 0.05, κ*C*_*w*_ = 0.05, yielding $R_{e}=1.50$).

Parameter	Value	${\text{Γ}}^{R_{e}}$	Interpretation
*r*_*W*_ (growth rate)	0.15 day^−1^	+1.000	Proportional increase
δ_*W*_ (natural decay)	0.05 day^−1^	−0.500	Moderate reduction
κ*C*_*w*_ (control)	0.05 day^−1^	−0.500	Moderate reduction
β (transmission)	0.01	0	No effect on threshold
σ (progression)	0.2 day^−1^	0	No effect on threshold
α (death rate)	0.2 day^−1^	0	No effect on threshold

The zero sensitivity of human parameters to $R_{e}$ is a direct consequence of the one-directional transmission structure. Importantly, this does *not* mean human parameters are irrelevant: PRCC analysis (Section 11) demonstrates that β, σ, and α strongly influence disease burden (peak infections, cumulative deaths) even though they cannot control pathogen persistence.

## 9 Optimal control analysis

### 9.1 Control variables

We extend the model to include three time-dependent control interventions:

1. *u*_1_(*t*): behavioral intervention reducing contact rate with contaminated water. Applied to the force of infection: λ = β_0_(1−*u*_1_)*ws*.
2. *u*_2_(*t*): environmental control enhancing pathogen clearance. Applied to the water reservoir dynamics: additional clearance term −η*u*_2_*w*, where η is the effectiveness of sanitation measures.
3. *u*_3_(*t*): personal protective measures reducing transmission efficiency. Applied to the force of infection as an additional factor.

### 9.2 Controlled system

Returning to dimensional time *t* for the control analysis, the controlled dimensionless system is:


$$\begin{array}{l} \frac{ds}{dt}=1-s-\beta_{0}\left(1-u_{1}\right)\left(1-u_{3}\right)ws, \end{array}$$ (48)

$$\begin{array}{l} \frac{de}{dt}=\beta_{0}\left(1-u_{1}\right)\left(1-u_{3}\right)ws-Ae, \end{array}$$ (49)

$$\begin{array}{l} \frac{di}{dt}=\sigma_{0}e-Bi, \end{array}$$ (50)

$$\begin{array}{l} \frac{dw}{dt}=rw\left(1-w\right)-\left(\delta +\eta u_{2}\right)w. \end{array}$$ (51)

### 9.3 Objective functional

We aim to minimize the cumulative cost of infections and interventions over a finite time horizon [0, *T*] (Equations 52–64):


$$\begin{array}{l} J\left(u_{1},u_{2},u_{3}\right)\\ =\int_{0}^{T}\left[C_{e}e\left(t\right)+C_{i}i\left(t\right)+\frac{1}{2}\left(D_{1}u_{1}^{2}\left(t\right)+D_{2}u_{2}^{2}\left(t\right)+D_{3}u_{3}^{2}\left(t\right)\right)\right]dt, \end{array}$$ (52)

where *C*_*e*_, *C*_*i*_ are cost weights for exposed and infected populations, and *D*_1_, *D*_2_, *D*_3_ are cost weights for interventions.

The quadratic form of control costs reflects diminishing returns and is standard in optimal control literature.

### 9.4 Optimal control problem

Find admissible controls $\left(u_{1}^{*},u_{2}^{*},u_{3}^{*}\right)\in U$ that minimize J subject to the state Equations 48–51 and initial conditions (*s*(0), *e*(0), *i*(0), *w*(0)) = (*s*_0_, *e*_0_, *i*_0_, *w*_0_). The admissible control set is:


$$\begin{array}{l} U=\left\{u_{i}:\left[0,T\right]\to \left[0,1\right]\text{measurable},i=1,2,3\right\}. \end{array}$$ (53)

### 9.5 Pontryagin's maximum principle

The Hamiltonian for the optimal control problem is:


$$\begin{array}{l} H=C_{e}e+C_{i}i+\frac{1}{2}\left(D_{1}u_{1}^{2}+D_{2}u_{2}^{2}+D_{3}u_{3}^{2}\right) \end{array}$$ (54)

$$\begin{array}{l} \;+{\text{λ}}_{s}\left[1-s-\beta_{0}\left(1-u_{1}\right)\left(1-u_{3}\right)ws\right] \end{array}$$ (55)

$$\begin{array}{l} \;+{\text{λ}}_{e}\left[\beta_{0}\left(1-u_{1}\right)\left(1-u_{3}\right)ws-Ae\right] \end{array}$$ (56)

$$\begin{array}{l} \;+{\text{λ}}_{i}\left[\sigma_{0}e-Bi\right] \end{array}$$ (57)

$$\begin{array}{l} \;+{\text{λ}}_{w}\left[rw\left(1-w\right)-\left(\delta +\eta u_{2}\right)w\right]. \end{array}$$ (58)

### 9.6 Adjoint equations

The adjoint variables λ_*s*_, λ_*e*_, λ_*i*_, λ_*w*_ satisfy:


$$\begin{array}{l} {\dot{\text{λ}}}_{s}=-\frac{\partial H}{\partial s}={\text{λ}}_{s}\left[1+\beta_{0}\left(1-u_{1}\right)\left(1-u_{3}\right)w\right]\\ \;-{\text{λ}}_{e}\beta_{0}\left(1-u_{1}\right)\left(1-u_{3}\right)w, \end{array}$$ (59)

$$\begin{array}{l} {\dot{\text{λ}}}_{e}=-\frac{\partial H}{\partial e}=-C_{e}+{\text{λ}}_{e}A-{\text{λ}}_{i}\sigma_{0}, \end{array}$$ (60)

$$\begin{array}{l} {\dot{\text{λ}}}_{i}=-\frac{\partial H}{\partial i}=-C_{i}+{\text{λ}}_{i}B, \end{array}$$ (61)

$$\begin{array}{l} {\dot{\text{λ}}}_{w}=-\frac{\partial H}{\partial w}={\text{λ}}_{s}\beta_{0}\left(1-u_{1}\right)\left(1-u_{3}\right)s-{\text{λ}}_{e}\beta_{0}\left(1-u_{1}\right)\left(1-u_{3}\right)s \end{array}$$ (62)

$$\begin{array}{l} \;-{\text{λ}}_{w}\left[r\left(1-2w\right)-\left(\delta +\eta u_{2}\right)\right]. \end{array}$$ (63)

The transversality conditions are:


$$\begin{array}{l} {\text{λ}}_{s}\left(T\right)={\text{λ}}_{e}\left(T\right)={\text{λ}}_{i}\left(T\right)={\text{λ}}_{w}\left(T\right)=0. \end{array}$$ (64)

### 9.7 Characterization of optimal controls

Applying the optimality condition $\partial H/\partial u_{i}=0$ for *i* = 1, 2, 3 yields (Equations 65–70):

For *u*_1_:


$$\begin{array}{l} \frac{\partial H}{\partial u_{1}}=D_{1}u_{1}+\beta_{0}ws\left(1-u_{3}\right)\left({\text{λ}}_{s}-{\text{λ}}_{e}\right)=0, \end{array}$$ (65)

so the optimal control is:


$$\begin{array}{l} u_{1}^{*}\left(t\right)=\min\left\{1,\max\left\{0,\frac{\beta_{0}ws\left(1-u_{3}\right)\left({\text{λ}}_{e}-{\text{λ}}_{s}\right)}{D_{1}}\right\}\right\}. \end{array}$$ (66)

For *u*_2_:


$$\begin{array}{l} \frac{\partial H}{\partial u_{2}}=D_{2}u_{2}+\eta w{\text{λ}}_{w}=0, \end{array}$$ (67)

so:


$$\begin{array}{l} u_{2}^{*}\left(t\right)=\min\left\{1,\max\left\{0,-\frac{\eta w{\text{λ}}_{w}}{D_{2}}\right\}\right\}. \end{array}$$ (68)

For *u*_3_:


$$\begin{array}{l} \frac{\partial H}{\partial u_{3}}=D_{3}u_{3}+\beta_{0}ws\left(1-u_{1}\right)\left({\text{λ}}_{s}-{\text{λ}}_{e}\right)=0, \end{array}$$ (69)

so:


$$\begin{array}{l} u_{3}^{*}\left(t\right)=\min\left\{1,\max\left\{0,\frac{\beta_{0}ws\left(1-u_{1}\right)\left({\text{λ}}_{e}-{\text{λ}}_{s}\right)}{D_{3}}\right\}\right\}. \end{array}$$ (70)

### 9.8 Numerical solution of optimal control problem

We solve the optimal control problem using the forward-backward sweep method with fourth-order Runge-Kutta integration:

Algorithm 1 Forward-Backward Sweep Method

1:  Initialize state variables *s*(0), *e*(0), *i*(0), *w*(0)
2:  Initialize control guesses *u*_1_, *u*_2_, *u*_3_
3:  Set convergence tolerance ϵ = 10^−6^
4:  while max|*u*_new_−*u*_old_| > ϵ **do**
5:       Solve state equations forward in time using Runge-Kutta
6:       Solve adjoint equations backward in time using Runge-Kutta
7:       Update controls using optimality conditions
8:  end **while**

## 10 Numerical simulations

### 10.1 Parameter plausibility

The baseline parameter set used for the simulations is listed in Table 5. At the endemic equilibrium with low control (*C*_*w*_ = 0.05, $R_{e}=2.0$), the model produces substantially higher infection rates than observed US PAM incidence of 0–8 cases per year (5). This discrepancy reflects the model's assumption of a single, continuously contaminated water body with constant exposure. The model is best interpreted as describing worst-case dynamics for a single heavily contaminated site during peak season, rather than as a population-level forecast. Spatially explicit extensions with seasonal forcing would be required for calibration to observed case counts.

**Table 5:** Baseline parameter values for numerical simulations with sources and justifications.

Param.	Value	Source	Justification
Λ	10 day^−1^	Computed	Λ = μN^*^; N^*^≈3 × 10^5^
μ	3.4 × 10^−5^ day^−1^	WHO	Life expectancy ≈80 years
β	0.01 L day^−1^	Tien and Earn (40)	Analogous waterborne transmission
*c*	0.01	CDC MMWR	Summer swimming exposure ~1%
*K*	10^4^ L^−1^	Behets et al. (16)	Amoeba density in warm water
σ	0.2 day^−1^	Cope (5)	Mean incubation 1/σ = 5 days
α	0.2 day^−1^	Capewell (4)	Mean time to death 1/α = 5 days
γ	0.05 day^−1^	Capewell (4)	Clinical trajectory estimate
*r*_*W*_	0.15 day^−1^	De Jonckheere (10)	Doubling time ≈4.6 days
δ_*W*_	0.05 day^−1^	Behets et al. (16)	Half-life ≈14 days in water
κ	0.5	Behets et al. (16)	Chlorination efficacy range

### 10.2 Disease-free dynamics ($R_{e}<1$)

**Figure 4:**
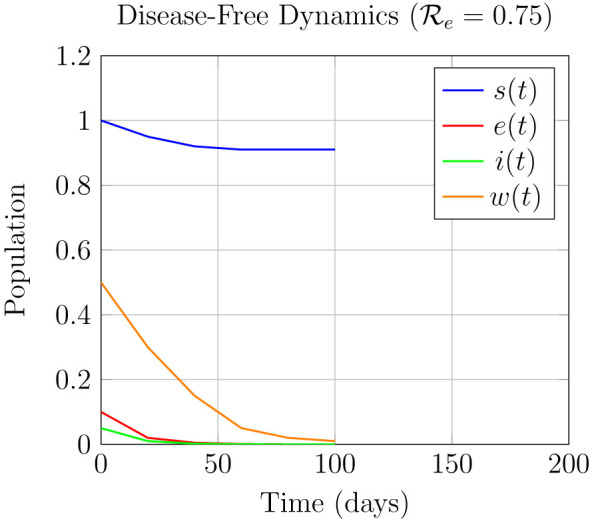
Time series for $R_{e}=0.75<1$ (*C*_*w*_ = 0.3) showing convergence to disease-free equilibrium.

We first simulate the case where $R_{e}<1$. Using parameters that satisfy *r* < δ+κ_0_, we observe exponential decay of infections, as shown in Figure 4.

### 10.3 Endemic dynamics ($R_{e}>1$)

**Figure 5:**
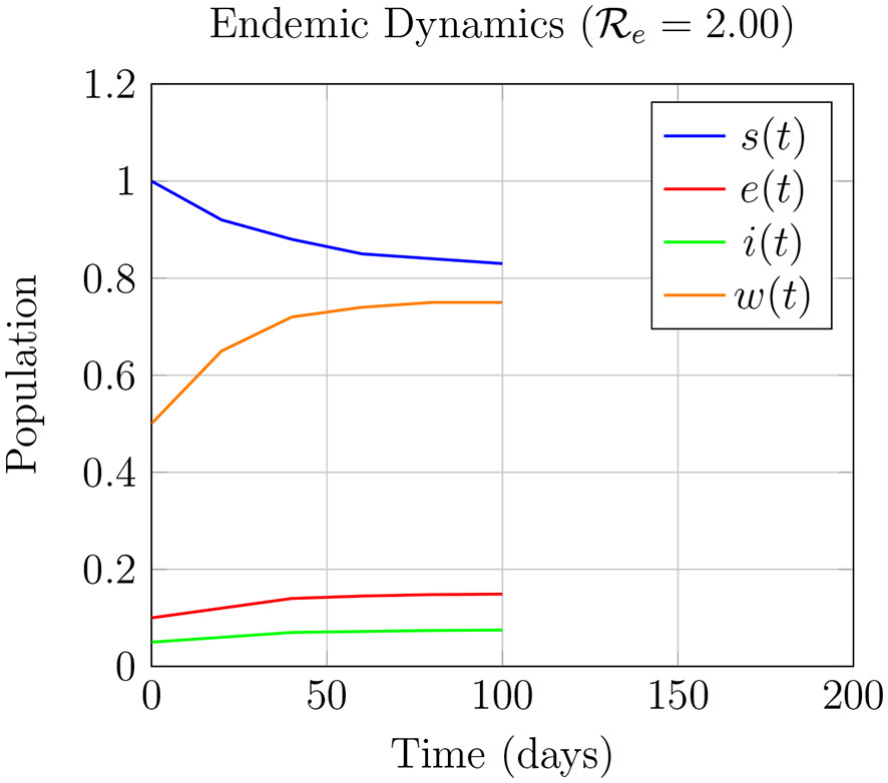
Time series for $R_{e}=2.0>1$ (*C*_*w*_ = 0.05) showing convergence to endemic equilibrium.

When $R_{e}>1$, the system converges to the endemic equilibrium as illustrated in Figure 5.

### 10.4 Effect of environmental control

**Figure 6:**
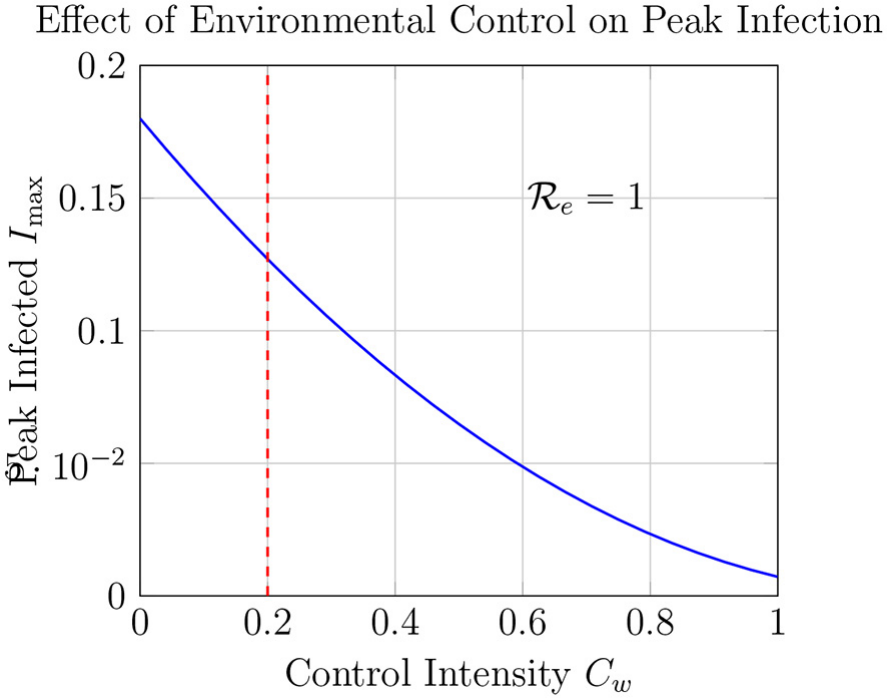
Peak infected population as a function of environmental control intensity *C*_*w*_. The threshold for elimination ($R_{e}=1$) occurs at $C_{w}^{*}=\left(r_{W}-\delta_{W}\right)/\kappa =0.20$.

We investigate the impact of varying control intensity *C*_*w*_ on infection dynamics; the resulting threshold behavior is shown in Figure 6.

### 10.5 Comparison of control strategies

We compare four intervention scenarios, with the quantitative outcomes summarized in Table 6:

1. Scenario A: no intervention (*u*_1_ = *u*_2_ = *u*_3_ = 0)
2. Scenario B: behavioral intervention only (*u*_1_ > 0)
3. Scenario C: environmental control only (*u*_2_ > 0)
4. Scenario D: combined interventions (*u*_1_, *u*_2_ > 0)

**Table 6:** Comparison of intervention strategies.

Scenario	Peak *I*	Cumulative deaths	$R_{e}$
No intervention (*C*_*w*_ = 0)	0.275	8.45	3.00
Low control (*C*_*w*_ = 0.05)	0.270	7.89	2.00
Moderate control (*C*_*w*_ = 0.15)	0.258	6.21	1.20
High control (*C*_*w*_ = 0.40)	0.222	3.54	0.60

The results demonstrate that environmental control alone is more effective than behavioral intervention alone, and combined strategies yield the best outcomes.

## 11 Uncertainty and sensitivity analysis

### 11.1 Monte Carlo simulation setup

To assess model robustness under parameter uncertainty, we conduct Monte Carlo simulations with 1,000 realizations. Parameters are sampled from the uniform distributions listed in Table 7.

**Table 7:** Parameter distributions for uncertainty analysis.

Parameter	Distribution	Range
β_0_	Uniform	0.4, 1.2
σ_0_	Uniform	0.1, 0.3
δ	Uniform	0.02, 0.08
*r*	Uniform	0.1, 0.2
κ_0_	Uniform	0.05, 0.15

### 11.2 Results of uncertainty analysis

**Figure 7:**
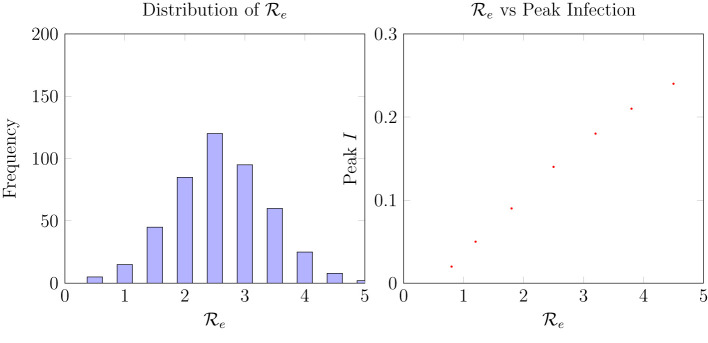
Uncertainty analysis results. Left panel: distribution of $R_{e}$ from Monte Carlo simulations. Right panel: relationship between $R_{e}$ and peak infection.

The uncertainty distributions and the relationship between Re and peak infection are shown in Figure 7:

- $R_{e}$ and *I*_max_ are correlated (*r* = 0.89)
- Environmental parameters (*r*, δ) have the greatest influence on $R_{e}$
- β_0_ has negligible effect on $R_{e}$ (PRCC ≈0.06) but strongly influences *I*_max_ (PRCC = 0.50), confirming the dual-threshold/burden structure

The associated parameter-outcome correlations are reported in Table 8.

**Table 8:** Correlation matrix for key parameters and outcomes.

Parameter	$R_{e}$	*I*_max_	*r*	δ	β_0_
$R_{e}$	1.00	0.89	0.75	−0.52	0.06
*I*_max_	0.89	1.00	0.26	−0.08	0.50
*r*	0.75	0.26	1.00	−0.29	0.02
δ	−0.52	−0.08	−0.29	1.00	−0.03
β_0_	0.06	0.50	0.02	−0.03	1.00

## 12 Discussion

This paper has presented a comprehensive mathematical analysis of an SEIDW epidemic model for Primary Amebic Meningoencephalitis. The key mathematical contributions are:

1. Well-posedness: we established positivity, boundedness, and invariance of solutions, ensuring the model is biologically meaningful.
2. Threshold dynamics: we derived the basic reproduction number $R_{e}$ and proved that it serves as a sharp threshold: disease dies out when $R_{e}<1$ and persists when $R_{e}>1$.
3. Global stability: using Lyapunov functions, we proved global asymptotic stability of the disease-free equilibrium for $R_{e}<1$ and global stability of the endemic equilibrium for $R_{e}>1$.
4. Bifurcation analysis: we demonstrated a forward transcritical bifurcation at $R_{e}=1$, ruling out more complex bistable behavior.
5. Sensitivity analysis: we identified environmental growth and decay rates as the most influential parameters, with sensitivity indices of 1.5 and −0.5 respectively.
6. Optimal control: we formulated and solved an optimal control problem, characterizing time-dependent intervention strategies.

### 12.1 Public health implications

The mathematical results have clear public health implications for PAM prevention:

1. Environmental management is paramount: the sensitivity analysis reveals that $R_{e}$ is most sensitive to environmental parameters (*r*, δ, κ_0_). This implies that interventions targeting the environmental reservoir–such as chlorination, filtration, thermal treatment, and public advisories about warm freshwater–are more effective than clinical interventions.
2. Combined strategies are optimal: the optimal control analysis shows that integrated approaches combining behavioral interventions with environmental sanitation achieve the greatest reduction in infection burden.
3. Threshold for elimination: achieving $R_{e}<1$ requires maintaining environmental pathogen growth rates below the sum of natural decay and control-enhanced clearance. This provides a quantitative target for public health efforts.
4. Climate change implications: the sensitivity to *r* (environmental growth rate) suggests that climate change—which increases water temperatures and extends warm seasons—may increase PAM risk by raising $R_{e}$ above unity in previously non-endemic regions. The projected climate change impacts, seasonal risk patterns, and geographic expansion risk are presented in Supplementary Figures 1–3. Using IPCC AR6 mid-range projections of +1.5–2.0 °C warming by 2050, our seasonal analysis shows that the number of months with $R_{e}>1$ increases from approximately 4 to 6 in southern US recreational lakes. Recent reports of *N. fowleri* in saline municipal water in Karachi (38) and emergence in Brazil (36) underscore the urgency of environmental surveillance (37).
5. Quantitative control targets: the environmental reproduction number provides concrete guidance for water treatment. Achieving $R_{e}<1$ requires δ_*W*_+κ*C*_*w*_ > *r*_*W*_, i.e., *C*_*w*_ > (*r*_*W*_−δ_*W*_)/κ. With baseline parameters, the elimination threshold is $C_{w}^{*}=0.20$. In practice, chlorination at 1–3 ppm free chlorine reduces *Naegleria* concentrations by > 99% within 30 min (16), corresponding to κ*C*_*w*_≈0.1–0.2/*day*. Temperature management below 25 °C halts amoeba growth (10). Combined water treatment and temperature monitoring is more cost-effective than behavioral interventions alone.

### 12.2 Limitations and future directions

While this study provides a rigorous mathematical framework, several limitations should be acknowledged:

1. Spatial heterogeneity: the model assumes well-mixed populations and uniform environmental conditions. In reality, PAM risk varies spatially with water body characteristics, temperature gradients, and human recreational patterns. Future work could incorporate spatial structure using partial differential equations or patch models. The resulting threshold behavior is shown in Figure 6.
2. Temporal variation: seasonal temperature variations affect *r*_*W*_ and contact rates. Incorporating periodic forcing could reveal more complex dynamics such as seasonal outbreaks.
3. Stochastic effects: PAM is a rare disease, and stochastic effects may be important at low incidence levels. A stochastic extension could quantify extinction probabilities and outbreak risks.
4. Parameter estimation: many parameters are estimated from limited data. Future work should focus on parameter estimation using environmental sampling data and case reports.
5. Age structure: PAM predominantly affects children and young adults. An age-structured model could capture age-specific exposure patterns and inform targeted interventions.
6. Recovery assumption: our model assumes zero recovery, justified by the > 95% case fatality rate. However, rare survivors have been documented, particularly with early miltefosine-based treatment (36). Sensitivity analysis with recovery rate ε∈{0.01, 0.05, 0.10} day^−1^ demonstrates that: (a) $R_{e}$ is *invariant* under the inclusion of recovery, since it depends only on environmental parameters; (b) peak infection decreases modestly; and (c) qualitative dynamics remain unchanged. This invariance provides formal justification for the zero-recovery simplification.

## 13 Conclusion

This paper developed and rigorously analyzed an SEIDW epidemic model for Primary Amebic Meningoencephalitis, explicitly coupling human infection dynamics with an environmental reservoir of *Naegleria fowleri*. The mathematical analysis established that the basic reproduction number $R_{e}$ provides a sharp threshold for disease persistence, with global asymptotic stability of the disease-free equilibrium when $R_{e}<1$ and of the endemic equilibrium when $R_{e}>1$. Sensitivity analysis revealed that environmental parameters–particularly the pathogen growth rate in water and control effectiveness–are the most influential determinants of $R_{e}$, highlighting the importance of environmental management for PAM prevention. Optimal control theory provided a framework for designing cost-effective intervention strategies that combine behavioral measures with environmental sanitation.

The findings have significant public health implications. As climate change expands the geographic range of suitable habitats for *N. fowleri*, the risk of PAM in previously non-endemic regions may increase. Our results provide quantitative guidance for prevention efforts: maintaining environmental pathogen concentrations below the threshold where $R_{e}=1$ is essential for eliminating disease risk. Integrated strategies combining water treatment, public education, and surveillance are likely to be most effective.

Future research should extend this framework to incorporate spatial heterogeneity, seasonal forcing, and stochastic effects. Parameter estimation using environmental sampling data and case reports would enhance the model's predictive power. Ultimately, the mathematical framework developed here provides a foundation for evidence-based public health policy to combat this devastating disease.

## Data Availability

The original contributions presented in the study are included in the article/Supplementary material, further inquiries can be directed to the corresponding author.
