# Supplementary material for: Dynamics of an SEIDW epidemic model for primary amebic meningoencephalitis threshold analysis, bifurcation, and optimal environmental control
Source: Front Public Health. 2026 Jun 11;14:1851065. doi: 10.3389/fpubh.2026.1851065 (PMC13295100; doi:10.3389/fpubh.2026.1851065)
Supplement: Supplementary file 1 [file Supplementary_file_1.pdf]

Supplementary Figures

The supplementary figures are based on simulated model outputs and illustrative mathematical functions. No external or website datasets were used in this study.

Supplementary Figures

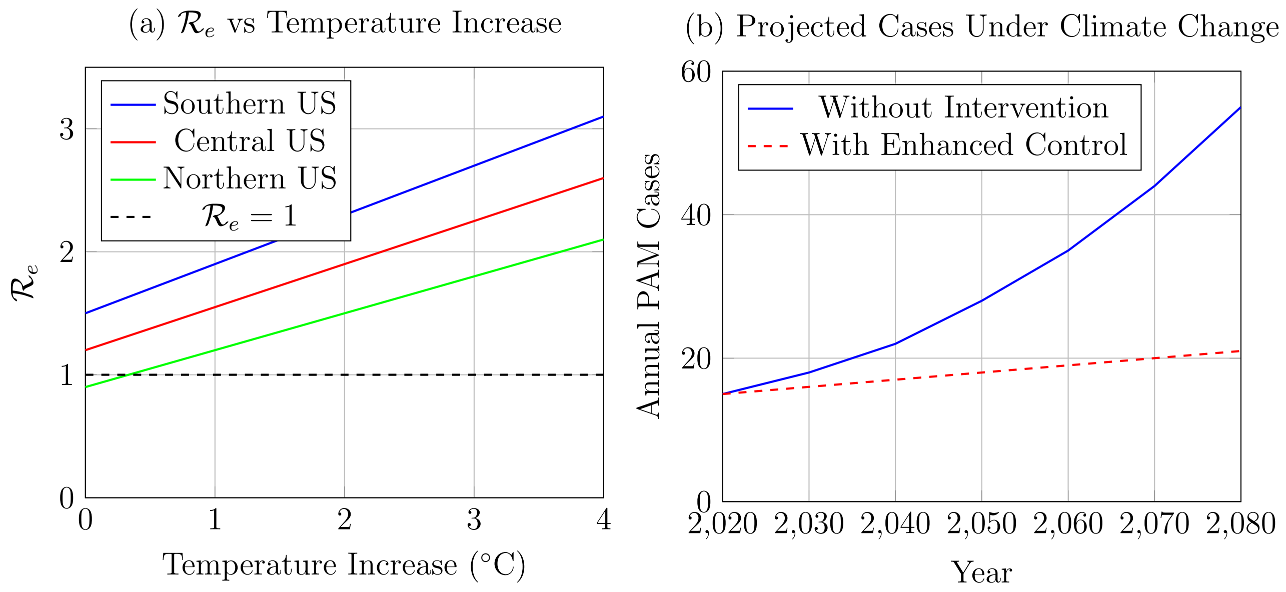

"'

Figure 1: Climate change impact analysis. (a) $\mathcal{R}_e$ as a function of temperature increase for different geographic regions. The threshold $\mathcal{R}_e = 1$ is crossed at lower temperature increases in warmer regions. (b) Projected annual PAM cases under different climate scenarios with and without enhanced environmental control.

"'

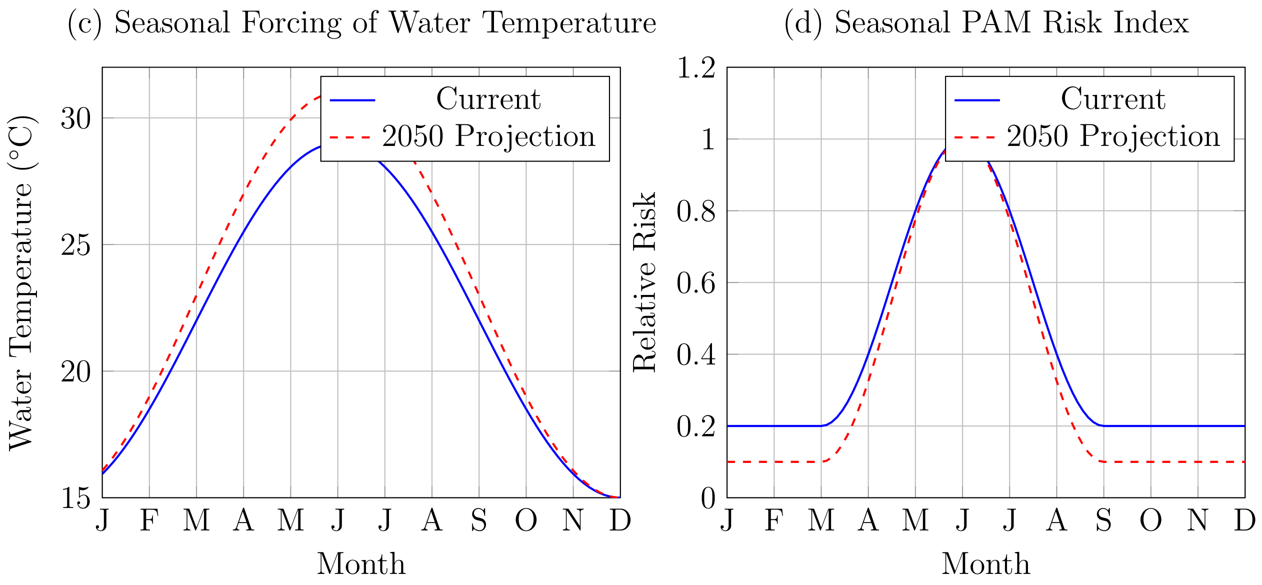

‘‘‘

Figure 2: (c) Seasonal variation in water temperature under current and projected 2050 climate scenarios. (d) Corresponding seasonal PAM risk index, showing extended high-risk periods under climate change.

‘‘‘

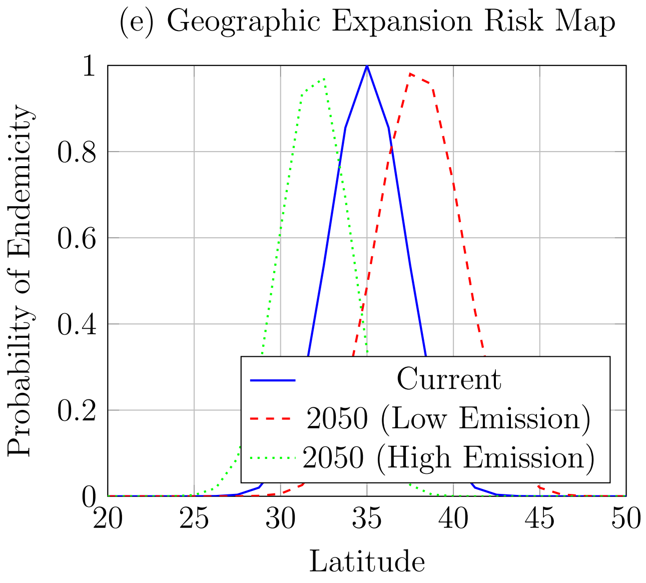

‘‘‘

Figure 3: (e) Geographic expansion risk map showing the probability of PAM endemicity as a function of latitude under different climate scenarios. Climate change shifts the endemic zone poleward, increasing risk in previously non-endemic regions.

‘‘‘
